# Ten-Year Outcomes in Colorectal Cancer—Competing Risks and Patient Vulnerability: A Prospective Multicenter Observational Study

**DOI:** 10.3390/jcm15114389

**Published:** 2026-06-05

**Authors:** Marilina García-Aranda, Desireé Martín-García, Janire Gallejones-Eskubi, Eloísa Urrechaga, Josefa Ferreiro, Vicente Portugal, Isabel Portillo, Marta Jiménez-Toscano, Maria Jose Legarreta, José María Quintana, Maximino Redondo, Urko Aguirre

**Affiliations:** 1 B-14 Research Group on Translational Research and Health Outcomes in Cancer and Chronic Diseases, Instituto de Investigación Biomédica de Málaga y Plataforma en Nanomedicina—IBIMA Plataforma BIONAND, 29590 Málaga, Spain; desiree.martin@ibima.eu (D.M.-G.); mredondo@uma.es (M.R.); 2 Research and Innovation Unit, Hospital Universitario Costa del Sol, 29602 Marbella, Spain; 3 Red de Investigación en Cronicidad, Atención Primaria y Promoción de la Salud (RICAPPS), Instituto de Salud Carlos III, 28029 Madrid, Spain; janire.gallejoneseskubi@bio-sistemak.eus (J.G.-E.); mariajose.legarretaolabarrieta@bio-sistemak.eus (M.J.L.); josemaria.quintanalopez@osakidetza.eus (J.M.Q.); 4 Surgical Specialties, Biochemistry and Immunology Department, Faculty of Medicine, University of Málaga, 29010 Málaga, Spain; 5 Research Unit, Osakidetza Basque Health Service, Barrualde-Galdakao Integrated Health Organisation, Galdakao-Usansolo Hospital, 48960 Galdakao, Spain; 6 Biosistemak Institute for Health System Research, Gran Vía, 1, 48001 Bilbao, Spain; 7 Clinical Laboratory, Galdakao-Usansolo Hospital, 48960 Galdakao, Spain; eloisamaria.urrechagaigartua@osakidetza.eus; 8 Biobizkaia Health Research Institute, Plaza Cruces s/n, 48903 Barakaldo, Spain; josefa.ferreiroquintana@osakidetza.eus (J.F.); vicente.portugalporras@osakidetza.eus (V.P.);; 9 Department of Oncology, Galdakao-Usansolo University Hospital, Osakidetza Basque Health Service, University of the Basque Country, Barrio Labeaga s/n, 48960 Galdakao, Spain; 10 University of the Basque Country, 48940 Leioa, Spain; 11 Department of Surgery, Galdakao-Usansolo University Hospital, Osakidetza Basque Health Service, Barrio Labeaga s/n, 48960 Galdakao, Spain; 12 Spanish Early-Onset Colorectal Cancer Consortium, Hospital del Mar, 08003 Barcelona, Spain; mjimeneztoscano@psmar.cat; 13 Surgery Department, Hospital del Mar, 08003 Barcelona, Spain

**Keywords:** colorectal cancer, long-term outcomes, cause-specific mortality, cancer survivorship, sex differences

## Abstract

**Background:** As survival after colorectal cancer (CRC) has improved, an increasing proportion of patients live beyond five years, making long-term outcomes increasingly relevant. In addition to cancer-related mortality, survivors remain at risk of death from other causes influenced by clinical and psychosocial vulnerabilities. **Methods:** We conducted a 10-year prospective cohort study including 838 patients with stage I–IV CRC treated in public hospitals in the Basque Country (Spain). Patients were recruited between November 2010 and December 2012 and followed for up to 10 years after surgery. Clinical, sociodemographic, lifestyle, and patient-reported outcomes were collected. Competing risk regression models (Fine-Gray) were used to estimate sub-distribution hazard ratios (sHRs) for CRC-specific and non-CRC mortality, stratified by tumor site and sex. **Results:** After 10 years, 40% of patients had died, with 66% of deaths attributable to CRC and 34% to other causes. CRC-specific mortality was mainly driven by tumor-related factors, including advanced stage (stage IV: sHR 7.18, *p* < 0.001) and residual disease after surgery (R1/R2: sHR 2.68; *p* < 0.001), with larger effect sizes observed in rectal cancer. In contrast, non-CRC mortality was associated with patient vulnerability, including age ≥75 years (sHR 3.57, *p* < 0.001), absence of adjuvant chemotherapy (sHR 5.59, *p* < 0.001), anemia, alcohol consumption, and poor functional status. Patients with rectal cancer and women reported poorer baseline quality of life. Sex-stratified analyses suggested differential patterns of vulnerability, with psychosocial and quality-of-life-related factors appearing more relevant in women, whereas lifestyle and clinical factors appeared more prominent in men. **Conclusions:** Long-term mortality in CRC reflects the interplay between tumor-related factors and patient vulnerability. Competing risk models allow a more accurate characterization of cause-specific outcomes and may help identify high-risk subgroups for tailored follow-up and management strategies.

## 1. Introduction

Cancer remains one of the major global challenges of the 21st century, accounting for nearly one in six deaths worldwide and 22.8% of mortality from non-communicable diseases [1]. Colorectal cancer (CRC), encompassing tumors of the colon and rectum, is the third most frequently diagnosed cancer worldwide and the second leading cause of cancer-related death, responsible for more than 665,000 deaths in 2022 [2]. That year, CRC accounted for 9.7% of cancer deaths from colon cancer and 3.5% from rectal or rectosigmoid tumors, ranking as the second leading cause of cancer mortality in men and the third in women [3]. In Spain, CRC is the most commonly diagnosed cancer in both sexes, representing 15.5% of all new cancer cases in 2024 [3], with five-year survival rate of 62.5% [4], leading to a growing population of long-term survivors [3]. These trends are driven by population aging, increased exposure to modifiable risk factors, and improvements in early detection and treatment, which have contributed to earlier-stage diagnosis and declining mortality [3]. In this regard, even though CRC screening programs have played a central role in reducing incidence and mortality through early detection and removal of precancerous lesions, participation rates remain suboptimal in many settings, and disparities in access and uptake continue to influence long-term outcomes [5]. Furthermore, although recent advances in artificial intelligence, particularly deep learning algorithms, have shown promising results in improving the accuracy of CRC detection and prognostic stratification from histopathological and clinical data [6,7], their integration into routine clinical practice and their impact on long-term patient outcomes remain limited.

In addition to its clinical impact, CRC poses a substantial economic and social burden on healthcare systems. In Europe alone, the total economic burden of colorectal cancer has been estimated at approximately €19 billion annually, with more than 60% attributable to indirect costs such as productivity losses and informal care [8]. Similarly, recent studies have highlighted the high direct and indirect costs associated with CRC management across different healthcare settings, further emphasizing the importance of optimizing long-term management strategies [9].

As the number of CRC survivors increases, understanding long-term survivorship outcomes beyond five years has become increasingly important. However, most studies evaluating CRC prognosis rely on conventional survival methods that do not adequately account for competing causes of death. This limitation is particularly relevant in aging populations, where non-cancer mortality represents a substantial proportion of long-term outcomes. For this reason, ignoring competing risks may lead to biased estimates of cancer-specific mortality and obscure the relative contribution of tumor-related and patient-related factors [10,11].

Current evidence on CRC prognosis has primarily focused on clinical and pathological characteristics. Surgical resection remains the cornerstone of treatment for resectable, non-metastatic CRC, with important differences between colon and rectal tumors due to anatomical and therapeutic factors that influence recurrence patterns and survival [12]. Postoperative surveillance strategies aim to detect recurrences at a curable stage, although commonly used biomarkers such as carcinoembryonic antigen (CEA) and carbohydrate antigen (CA19-9) have limited sensitivity and specificity [13]. While inflammatory markers such as the neutrophil-to-lymphocyte ratio (NLR) and the monocyte-to-lymphocyte ratio (MLR) have shown prognostic value in short- and mid-term outcomes [14], recent evidence suggests that intensive surveillance strategies may not improve long-term mortality, supporting more selective and risk-adapted approaches [15].

In addition, most prognostic models have insufficiently integrated non-clinical determinants. Survivorship is increasingly shaped by sociodemographic, lifestyle, and psychological factors, including quality of life, comorbidity, fatigue, anxiety, and depression, which may influence treatment adherence and survival [16,17,18]. Biological sex also plays a relevant role in CRC outcomes, affecting incidence, mortality, comorbidity burden, and psychosocial profiles, with women often experiencing greater impairments in health-related quality of life despite lower incidence rates [19]. However, the long-term influence of these factors, particularly beyond the first five years after diagnosis, remains insufficiently explored [20].

We have previously identified several short- and mid-term prognostic factors in CRC, including advanced stage, comorbidity, residual disease after surgery (R1/R2), poor preoperative health status and reduced quality of life [21]. Nevertheless, evidence on long-term outcomes remains limited, and current guidelines provide little direction beyond five years of follow-up, despite evidence that a proportion of recurrences occur later [22]. Moreover, prior analyses have not consistently distinguished between CRC-specific and non-CRC mortality, limiting the interpretation of long-term risk [23].

To address this gap, we conducted a 10-year prospective cohort study of patients with CRC, applying competing risk regression models to distinguish between CRC-specific and non-CRC mortality. By integrating clinical, pathological, sociodemographic, and patient-reported variables, and by exploring heterogeneity according to tumor location and sex, this study aims to provide a more comprehensive understanding of the determinants of long-term outcomes in CRC.

## 2. Materials and Methods

### 2.1. Study Design, Scope, and Period of Study

This study is part of the national CARESS-CCR cohort, a multicenter, prospective, observational cohort study [24]. Between June 2010 and December 2012, the CARESS-CCR cohort enrolled 3915 individuals from 22 public hospitals across nine provinces in Spain. All participants had access to universal healthcare through the Spanish public health system, which provides free primary and hospital care. Of the total recruited, 2749 patients met the inclusion criteria and were formally included in the cohort.

Patient recruitment in the Basque Country started in November 2010. The present analysis was restricted to 898 patients recruited from eight hospitals in the Basque Country: Hospital Galdakao-Usansolo, Hospital Universitario Araba, Hospital Universitario Basurto, Hospital Universitario Cruces, Hospital Donostia, Hospital Bidasoa, Hospital de Mendaro, and Hospital de Zumarraga. After excluding 60 patients who died from other neoplasms, the final analytical sample comprised 838 patients who were followed for a planned period of 10 years after surgery. Among them, 220 died from CRC and 114 from other causes.

### 2.2. Inclusion and Exclusion Criteria

Eligible participants were patients diagnosed with colon cancer (>15 cm from the anal margin) or rectal cancer (≤15 cm from the anal margin), stages I–IV, who underwent surgery with curative intent, either elective or urgent, between 2010 and 2012. Exclusion criteria included in situ tumors, unresectable disease, multiple synchronous primary tumors, and appendiceal neoplasms. Patients whose mental or physical conditions precluded completion of the study questionnaires, as well as those who were terminally ill at diagnosis, were also excluded.

### 2.3. Data Collection and Variables

Clinical data were collected from medical records and institutional databases by trained reviewers using a standardized protocol to ensure consistency across centers. Patients were prospectively and consecutively enrolled at each participating hospital. Pathology diagnosis was confirmed by histological examination of biopsy samples obtained via colonoscopy or, when applicable, at the time of surgery.

Clinical and patient-reported outcome data were collected at baseline (prior to surgery) and at 1, 2, 3, 5, 8, and 10 years after surgery. Survival status at 10 years was obtained from hospital databases, patient and family questionnaires, and the Spanish National Death Index. Cause-specific mortality was obtained from the Mortality Register of the Basque Government Department of Health.

#### 2.3.1. Independent Variables

Variables were grouped into the following domains:

- Sociodemographic variables: age (<75 or ≥75 years), sex, educational level, marital status, and employment status.
- Clinical and lifestyle variables: body mass index (BMI, categorized according to WHO criteria), family history of neoplasia, smoking status (non-smoker, current, former), alcohol consumption (yes/no), comorbidity (Charlson Comorbidity Index [25], diabetes status, and symptoms at diagnosis (categorized as asymptomatic, mild or moderate/severe).
- Preoperative laboratory variables: neutrophil-to-lymphocyte ratio (NLR, non-pathological <5, or pathological ≥5), platelet-to-lymphocyte ratio (PLR, non-pathological <210, or pathological ≥210), systemic Inflammatory Index (SII, non-pathological <390, pathological ≥390), hemoglobin (pathological <13 g/dL in men and <12 g/dL in women, non-pathological ≥13 g/dL in men and ≥12 g/dL in women).
- Tumor markers: carcinoembryonic antigen (CEA, normal 0–5 ng/mL, abnormal >5 ng/mL), carbohydrate antigen 19-9 (Ca 19-9, normal <37 U/mL, abnormal >37 U/mL).
- Tumor characteristics: tumor location (right colon: appendix, caecum, ascending colon, hepatic flexure, and transverse colon, left colon: splenic flexure, descending colon, sigmoid and rectosigmoid, or rectum), pathological tumor-node-metastasis (pTNM) stage according to the AJCC 7th edition 2010 [26]. For subgroup analyses, right- and left-sided colon tumors were grouped as colon cancer and compared with rectal cancer.
- Surgical variables: ASA status [27], urgency of surgery, surgical approach, residual tumor status (R0: Complete absence of the tumor, R1: Microscopic presence of the tumor, R2: Macroscopic presence), and postoperative infectious complications.
- Oncological treatment: Neoadjuvant chemotherapy, adjuvant chemotherapy, radiotherapy).
- Patient-reported outcomes measures (PROMs): anxiety and depression assessed using the Hospital Anxiety and Depression Scale (HADS) [28,29], and health-related quality of life assessed using the European Organization for Research and Treatment of Cancer Quality of Life Questionnaire-C30 (EORTC QLQ-C30) [30,31].

Continuous variables were categorized using clinically established or commonly used cut-offs to facilitate interpretability and potential applicability in routine clinical practice.

#### 2.3.2. Outcome Variables

The primary outcomes were CRC-specific mortality and mortality from causes unrelated to CRC, treated as competing events.

### 2.4. Laboratory Methods

Peripheral blood samples were analyzed in the central laboratories of the participating hospitals within 24 h of blood collection using a Beckman Coulter LH750 hematology analyzer, following standardized protocols and routine clinical practice.

Based on peripheral blood cell counts, three systemic inflammatory markers were calculated: the SII, defined as neutrophils/lymphocyte × platelets; NLR (neutrophils/lymphocytes), and PLR (platelets/lymphocytes). All ratios were calculated using absolute cell counts (10^3^/µL).

### 2.5. Statistical Analysis

Descriptive statistics were computed for all study variables. Continuous variables were summarized using means and standard deviations (SD) or medians and interquartile ranges (IQR), as appropriate, while categorical variables were presented as frequencies and percentages.

The primary outcomes were CRC-specific mortality and mortality from other causes, considered as competing events. Associations between exposure variables and outcomes were initially explored using chi-square test or Fisher’s exact test for categorical variables and the Wilcoxon rank-sum test for continuous variables. Analyses were stratified by tumor location and biological sex.

Cumulative incidence functions were estimated to describe cause-specific mortality over time. Given the presence of competing events, Fine-Gray sub-distribution hazard models were used to identify factors associated with CRC-specific and non-CRC mortality, providing estimates of sub-distribution hazard ratios (sHRs) with 95% confidence intervals (CIs). Multivariable models were constructed using a modified backward elimination procedure (Allen-Cady), including variables with a *p*-value < 0.20 in univariate analyses [32]. Model performance was assessed using Harrell’s C-index, and internal validation was conducted through bootstrap resampling with 2000 replicates. All statistical analyses and figure generation were performed using R software (version 4.1.1).

## 3. Results

### 3.1. Study Population and Mortality Outcomes

#### Baseline Characteristics and Factors Associated with Mortality

The study cohort included 838 patients, of whom 504 (60.1%) were alive after 10 years of follow-up. Among the 334 deaths, 220 were attributable to CRC, and 114 to other causes. Baseline characteristics according to cause-specific mortality are presented in Table 1 and Appendix A Table A1.

Several sociodemographic, clinical, and treatment-related variables were significantly associated with mortality at 10 years.

Older age (≥75 years) was associated with higher overall mortality, particularly from non-CRC causes, whereas younger patients showed a higher proportion of CRC-specific deaths (*p* < 0.001). Unemployment and higher comorbidity burden were also associated with increased mortality, especially from non-CRC causes.

Among clinical and tumor-related variables, advanced pathological stage, rectal tumor location, and incomplete tumor resection (R1/R2) were strongly associated with CRC-specific mortality (all *p* < 0.001). Elevated inflammatory markers (NLR ≥ 5) were also associated with increased CRC-related deaths.

Regarding treatment, the absence of adjuvant chemotherapy was associated with higher overall mortality, particularly from non-CRC causes (*p* < 0.001).

### 3.2. Competing Risk Models for 10-Year Mortality

In multivariable competing risk models, distinct patterns emerged for CRC-specific and non-CRC mortality (Appendix A Table A2).

#### 3.2.1. CRC-Specific Mortality

CRC-specific mortality was primarily driven by tumor-related factors. Advanced disease stage was the strongest predictor, with stage IV associated with more than a seven-fold increase in risk compared with early-stage (sHR 7.18; 95% CI: 4.77–10.82; *p* < 0.001). Residual tumor after surgery (R1/R2) and poor preoperative health status (ASA IV) were also strongly associated with increased mortality (sHR 2.68; 95% CI: 1.54–4.67; *p* < 0.001). Additional factors included stage III disease (sHR 2.52; 95% CI: 1.82–3.49; *p* < 0.001), alcohol consumption (sHR 2.06; 95% CI: 1.45–2.92; *p* < 0.001), older age (≥75 years) (sHR 1.92; 95% CI: 1.43–2.58; *p* < 0.001), and pathological hemoglobin levels (sHR 1.41; 95% CI: 1.06–1.88; *p* = 0.01), all of which were independently associated with higher CRC-specific mortality. The model showed good discriminative performance (C-index: 0.76; 95% CI: 0.71–0.78).

#### 3.2.2. Mortality from Other Causes

In contrast, mortality from non-CRC causes was mainly associated with patient-related vulnerability. The absence of adjuvant chemotherapy was the strongest predictor (sHR 5.59; 95% CI: 2.65–11.81; *p* < 0.001), followed by older age (≥75 years) (sHR 3.57; 95% CI: 2.19–5.08; *p* < 0.001), poor preoperative status (ASA IV) (sHR 2.68; 95% CI: 1.37–5.26; *p* = 0.004), alcohol consumption (sHR 2.51; 95% CI: 1.48–4.27; *p* < 0.001), and pathological hemoglobin levels (sHR 2.32; 95% CI: 1.45–3.72; *p* < 0.001). Unemployment at diagnosis was also independently associated with increased non-CRC mortality (sHR 2.26; 95% CI: 1.03–4.98; *p* = 0.04). The model demonstrated strong discriminative ability (C-index 0.85; 95% CI: 0.80–0.86).

### 3.3. Competing Risk Models According to Tumor Location

#### 3.3.1. Baseline Differences

Baseline characteristics differed between patients with colon and rectal tumors (Appendix A Table A3). Patients with rectal cancer were older at diagnosis, presented with more severe symptoms, and had worse surgical outcomes, including higher rates of incomplete resection (R1/R2) and postoperative complications. They also more frequently received neoadjuvant treatment and reported lower baseline quality of life.

In contrast, patients with colon cancer more often had a family history of CRC, higher inflammatory marker levels, and a higher proportion of non-CRC-related deaths.

#### 3.3.2. CRC-Specific Mortality

CRC-specific mortality was primarily driven by tumor-related factors in both colon and rectal cancer, although effect sizes were consistently larger in rectal tumors (Appendix A Table A4 and Table A5).

In colon cancer, stage IV disease was the strongest predictor (sHR 4.92; 95% CI: 2.46–9.83; *p* < 0.001), followed by postoperative infectious complications (sHR 2.52; 95% CI: 1.46–4.34; *p* < 0.001), and residual tumor after surgery (R1/R2) (sHR 2.05; 95% CI: 1.17–3.57; *p* = 0.01). Male sex and age ≥75 years were also independently associated with increased risk. The model showed good discrimination (C-index 0.73; 95% CI: 0.65–0.78).

In rectal cancer, the magnitude of association was substantially higher, with stage IV disease associated with a more than fourteenfold increase in risk (sHR 14.22; 95% CI: 7.52–26.88; *p* < 0.001), followed by residual tumor (sHR 5.05; 95% CI: 2.81–9.09; *p* < 0.001) and absence of adjuvant chemotherapy (sHR 3.11; 95% CI: 1.05–9.27; *p* = 0.04). Additional factors reflecting systemic vulnerability, including poor preoperative status (ASA IV), pathological NLR, anemia, alcohol consumption, and older age, were also independently associated with CRC mortality. The model showed good discrimination (C-index 0.81; 95% CI: 0.75–0.83).

#### 3.3.3. Non-CRC Mortality

In contrast, non-CRC mortality was mainly associated with patient-related vulnerability across both tumor locations.

In colon cancer, age ≥75 years was the strongest predictor (sHR 8.29; 95% CI: 3.07–22.34; *p* < 0.001), followed by absence of adjuvant chemotherapy (sHR 7.03; 95% CI: 1.58–31.14; *p* = 0.01), and alcohol consumption (sHR 3.70; 95% CI: 1.31–10.46; *p* = 0.014). The model demonstrated excellent discrimination (C-index 0.87; 95% CI: 0.78–0.90).

In rectal cancer, a broader range of vulnerability-related factors contributed to non-CRC mortality, including age (≥75 years) (sHR 4.09; 95% CI: 2.53–6.63; *p* < 0.001), absence of adjuvant chemotherapy (sHR 4.24; 95% CI: 1.90–9.48; *p* < 0.001), comorbidity burden, poor preoperative status (ASA IV), alcohol consumption, and anemia. The model also showed strong discrimination (C-index 0.85; 95% CI: 0.80–0.87).

### 3.4. Competing Risk Models Stratified by Biological Sex

#### 3.4.1. Baseline Differences

Baseline characteristics also differed according to biological sex. Men more frequently had lower educational attainment, were married or partnered, were employed, and reported higher rates of former smoking and alcohol consumption. Women, in contrast, showed a higher prevalence of pathological hemoglobin, family history of CRC, comorbidity burden, and anxiety/depression, with a greater proportion exceeding the clinical threshold on HADS-A and HADS-D. After 10 years of follow-up, a higher proportion of women remained alive, although men reported better overall quality of life.

Distinct patterns of mortality were observed according to biological sex (Appendix A Table A6 and Table A7).

#### 3.4.2. CRC-Specific Mortality

CRC-specific mortality was primarily driven by tumor-related factors in both sexes, although some differences were observed in the contribution of patient-related variables.

In women, advanced disease was the strongest predictor (stage IV: sHR 10.39; 95% CI: 5.14–21.25; *p* < 0.001), followed by incomplete tumor resection (R1/R2) (sHR 2.49; 95% CI: 1.18–5.23; *p* = 0.01), pathological hemoglobin (sHR 2.06; 95% CI: 1.18–3.61; *p* = 0.01), stage III disease (sHR 2.08; 95% CI: 1.08–4.00; *p* = 0.02). Notably, psychosocial factors such as anxiety or depression were also independently associated with increased CRC mortality (sHR 1.76; 95% CI: 1.01–3.07; *p* = 0.04). The model showed good discrimination (C-index 0.74; 95% CI: 0.66–0.79).

In men, CRC mortality was also strongly associated with tumor burden, with stage IV disease as the main predictor (sHR 5.29; 95% CI: 3.16–8.86; *p* < 0.001), followed by residual tumor after surgery (R1/R2) (sHR 3.26; 95% CI: 2.05–5.17; *p* < 0.001) and stage III disease (sHR 2.69; 95% CI: 1.82–3.98; *p* < 0.001). Additional factors included older age (≥75 years), family history of neoplasia, and alcohol consumption. The model showed good discrimination (C-index 0.74; 95% CI: 0.68–0.77).

#### 3.4.3. Non-CRC Mortality

Non-CRC mortality was mainly associated with patient-related vulnerability in both sexes, although patterns differed in magnitude and breadth.

In women, age ≥75 years was the strongest predictor (sHR 14.98; 95% CI: 5.48–40.91; *p* < 0.001), followed by alcohol consumption (sHR 8.85; 95% CI: 1.86–42.06; *p* = 0.006), absence of adjuvant chemotherapy (sHR 4.93; 95% CI: 1.48–16.43; *p* = 0.009), and unemployment (sHR 2.93; 95% CI: 1.08–7.95; *p* = 0.03). The model showed strong discrimination (C-index 0.88; 95% CI: 0.79–0.92).

In men, the strongest predictor was absence of adjuvant chemotherapy (sHR 7.19; 95% CI: 2.83–18.31; *p* < 0.001), followed by poor preoperative status (ASA IV) (sHR 3.46; 95% CI: 1.67–7.16; *p* < 0.001), age ≥75 years (sHR 3.08; 95% CI: 1.82–5.19; *p* < 0.001), pathological hemoglobin (sHR 2.52; 95% CI: 1.48–4.30; *p* < 0.001), and alcohol consumption (sHR 2.12; 95% CI: 1.21–3.71; *p* = 0.008). The model showed excellent discrimination (C-index 0.84; 95% CI: 0.79–0.87).

#### 3.4.4. Sex Differences in Long-Term Mortality

**Figure 1:**
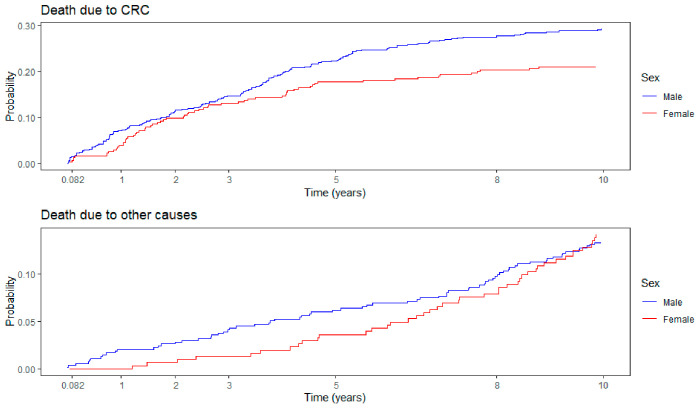
Cumulative incidence curves illustrating the probability of death due to colorectal cancer (CRC) and other causes over a 10-year follow-up, stratified by sex. The upper panel displays CRC-specific mortality, where men consistently demonstrate a higher cumulative incidence of death compared to women across the entire follow-up period (*p* = 0.01). The lower panel illustrates mortality due to causes other than CRC, with sex-related differences also observed, though less marked. Men show a slightly higher probability of death from other causes, particularly during the early years of follow-up (*p* = 0.005).

Figure 1 shows cumulative incidence curves over 10 years. Men exhibited a consistently higher probability of CRC-specific mortality, particularly in later follow-up. For non-CRC mortality, differences were more pronounced early after diagnosis, with a slightly higher risk in men that attenuated over time (Figure 1).

## 4. Discussion

To our knowledge, this is one of the few long-term prospective studies applying competing risk models to simultaneously evaluate CRC-specific and non-CRC mortality while integrating clinical, sociodemographic, lifestyle, and patient-reported variables. This approach provides a more nuanced understanding of long-term survivorship beyond conventional survival analyses. These findings are particularly relevant in the context of cancer survivorship, where long-term outcomes are shaped not only by tumor-related factors but also by patient-related and psychosocial vulnerability.

Our findings show that long-term outcomes are shaped by two distinct but overlapping domains: tumor-related factors, which primarily drive CRC mortality, and patient-related vulnerability, which largely determines mortality from other causes. Importantly, several variables identified in this study may be better understood as markers of underlying vulnerability, reflecting frailty, comorbidity burden, or social disadvantage, rather than direct causal determinants of mortality. This integrated perspective highlights the heterogeneity of CRC survivorship beyond the conventional five-year follow-up period and underscores the importance of appropriate analytical approaches for interpreting long-term outcomes.

### 4.1. Prognostic Factors and Competing Risks

Consistent with previous evidence, CRC-specific mortality was strongly associated with tumor burden, particularly advanced TNM stage and residual disease after surgery [21]. Additional contributions from factors such as elevated NLR, and treatment-related variables reflect the interaction between tumor aggressiveness and host response [12,33].

Unexpectedly, descriptive analyses suggested a higher proportion of CRC-specific mortality among younger and employed patients (Table 1). Although studies on early-onset CRC have described more aggressive tumor biology and diagnostic delays in patients under 50 years [34], these patterns were not confirmed in the multivariable models, where older age remained independently associated with increased CRC-specific mortality. Therefore, these descriptive findings should be interpreted with caution, as they may reflect confounding factors or differences in case-mix rather than true prognostic effects.

In contrast, non-CRC mortality was primarily associated with markers of vulnerability, including advanced age, comorbidity, unemployment, and absence of adjuvant chemotherapy, which are consistent with frailty and treatment underutilization [35]. Importantly, higher depression scores were associated with non-CRC mortality, in line with meta-analytic evidence showing that depression independently increases all-cause mortality among CRC survivors [35].

Competing risk regression further confirmed that tumor-related variables, particularly advanced stage (TNM III–IV) and residual disease after surgery (R1/R2), were the strongest predictors of CRC-specific mortality. In contrast, non-CRC mortality was mainly associated with patient-related vulnerability, particularly absence of adjuvant chemotherapy, older age, unemployment, poor preoperative status (ASA IV), alcohol consumption, and pathological hemoglobin levels [36], which may reflect underlying frailty, comorbidity burden, or treatment selection processes. The strong influence of unemployment at diagnosis further supports this interpretation, particularly in older individuals, where it may act as a proxy of poor functional or socioeconomic status.

Several variables, including age, ASA status, alcohol consumption and hemoglobin levels, were shared predictors of both CRC-specific and non-CRC mortality, although with different magnitudes of effect. This finding highlights the value of competing risk models, which account for the influence of competing events and allow more accurate estimation of cause-specific risks and the impact of comorbidities and lifestyle factors over extended follow-up periods [10]. The stronger effect of age on non-CRC mortality compared with CRC mortality further supports the concept that tumor aggressiveness and patient vulnerability contribute differently to long-term outcomes, highlighting the need for integrated survivorship strategies that combine oncological, physiological, and lifestyle factors especially targeting modifiable behaviors.

### 4.2. Tumor Location and Long-Term Outcomes

Our results confirm the prognostic heterogeneity between colon and rectal cancer [37,38], with patients with rectal tumors presenting a more adverse clinical profile at diagnosis, including older age, more severe symptoms, worse surgical outcomes, and lower quality of life, while colon cancer patients more frequently showed family history and higher inflammatory markers. Indeed, although advanced TNM stage, residual tumor, and older age were predictors in both locations, effect sizes appeared to be greater in rectal cancer (stage IV HR 14.22 vs. HR 4.92 in colon; R1/R2 HR 5.05 vs. HR 2.05), likely reflecting the distinct embryological origins, molecular pathways, and surgical challenges associated with rectal tumors [37,39,40].

Interestingly, while ASA status, anemia, inflammatory markers, alcohol consumption, and absence of adjuvant chemotherapy were more strongly associated with outcomes in rectal cancer, emerging as indicators of systemic vulnerability and treatment intensity, male sex and postoperative infectious complications were associated with CRC-specific mortality in colon cancer. Despite the increasing interest in emerging biomarkers, such as butyrylcholinesterase, as predictors of postoperative complications and patient vulnerability [41], surgical site infections remain among the most common complications following colorectal surgery and are associated with increased morbidity, prolonged hospital stay, higher healthcare costs, and, in some cases, worse survival outcomes [42]. In this regard, previous work from our group identified postoperative complications as relevant factors associated with prolonged hospital stay after colon cancer surgery [43].

Rectal resections, often requiring total mesorectal excision and sometimes preceded by neoadjuvant therapy, are associated with higher perioperative morbidity, increased bacterial load, and confined pelvic anatomy [37,39]. As a result, infectious complications are more frequent in rectal surgery and may represent a common event with limited discriminatory value for long-term prognosis. In contrast, colectomy is technically less demanding and associated with lower complication rates; therefore, postoperative infection may serve as a stronger marker of underlying vulnerability or surgical difficulty in colon cancer. Supporting this, a large series of 1083 patients demonstrated that postoperative infections were associated with poorer cancer-specific survival but not with overall survival, with their effect being more evident in subgroups defined by demographic and tumor-related factors [44]. Furthermore, a recent meta-analysis highlighted that postoperative infections may not only reflect surgical complexity but also underlying host-related vulnerability [45], which could explain their stronger prognostic role in colon cancer than in rectal cancer, where multiple competing risk factors already dominate long-term outcomes.

In rectal cancer, indicators of systemic vulnerability and treatment intensity were more strongly associated with outcomes, with poor ASA status, pathological hemoglobin levels, elevated NLR, and alcohol consumption emerging as significant predictors, alongside the absence of adjuvant chemotherapy. These results suggest that rectal cancer patients often enter treatment with reduced physiological reserve and are exposed to more intensive multimodal regimens, magnifying the impact of baseline health and treatment tolerance [36,37]. The prognostic significance of not receiving adjuvant chemotherapy in this subgroup may also indicate treatment intolerance due to frailty or comorbidities, or underutilization of therapy in older or vulnerable patients [36,46].

The distinct diagnostic profile of rectal cancer, where disease-specific mortality appears more closely linked to systemic vulnerability and treatment intensity, extended to non-cancer mortality as well. While advanced age (≥75 years), alcohol consumption, and lack of adjuvant chemotherapy were predictors shared with colon cancer, additional baseline characteristics such as elevated Charlson comorbidity index, poor ASA status, and anemia were independently associated with non-cancer deaths in rectal cancer. Notably, the effect of age was sharper in colon cancer, suggesting that elderly patients with colonic tumor may carry a higher overall frailty burden or reduced physiological reserve, leading to increased susceptibility to competing causes of death. By contrast, rectal cancer patients appeared more medically complex at baseline, with long-term outcomes shaped not only by tumor progression but also by comorbidity and functional status.

**Figure 2:**
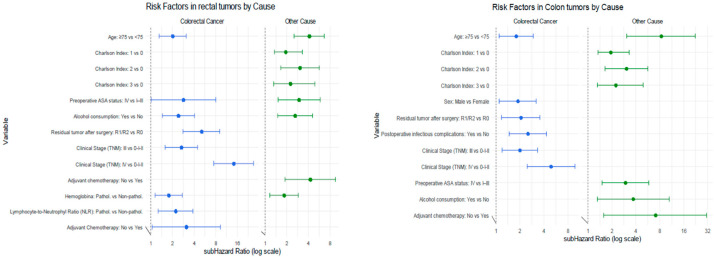
Competing Risk Model for 10-Year Mortality in Colorectal Cancer Patients by Tumor Location (Colon vs. Rectum). The forest plot illustrates distinct patterns of risk factors associated with long-term mortality based on tumor location. Blue lines represent CRC-specific mortality and green lines represent non-CRC mortality.

Altogether, our findings underscore the need for location-specific prognostic assessment and survivorship strategies. Colon cancer outcomes appear more sensitive to perioperative and demographic factors, while rectal cancer prognosis is strongly influenced by systemic health, comorbidity, and treatment tolerance (Figure 2), highlighting the importance of comprehensive baseline evaluation and site-tailored care planning. Future studies should explore whether targeted prehabilitation and optimization strategies can mitigate these site-specific vulnerabilities.

### 4.3. Sex-Specific Patterns in Mortality

Sex-stratified analyses revealed distinct and clinically relevant risk profiles. While advanced stage and residual disease remained dominant predictors of CRC-specific mortality in both sexes, their impact appeared to be stronger in women, consistent with previous reports of delayed diagnosis and differences in clinical management [47].

In men, CRC mortality was mainly associated with classical clinical and lifestyle factors, including alcohol consumption, older age, and family history of neoplasia. In contrast, in women, pathological hemoglobin levels and psychosocial factors such as anxiety and depression were independently associated with CRC mortality, highlighting a potentially greater role of psychosocial vulnerability. These findings are consistent with previous analyses showing that depressive symptoms are associated with increased long-term mortality in CRC patients [18], reinforcing the need for long-term psychosocial monitoring and interventions to improve both quality of life and survival outcomes, particularly in women.

For non-CRC mortality, men showed a broader risk profile, including comorbidity, anemia, alcohol consumption and lack of adjuvant chemotherapy, suggesting a higher burden of vulnerability. In women, fewer predictors were identified, but some had stronger effects, particularly age, alcohol consumption, and unemployment, suggesting that social determinants may play a more prominent role in long-term outcomes [48].

**Figure 3:**
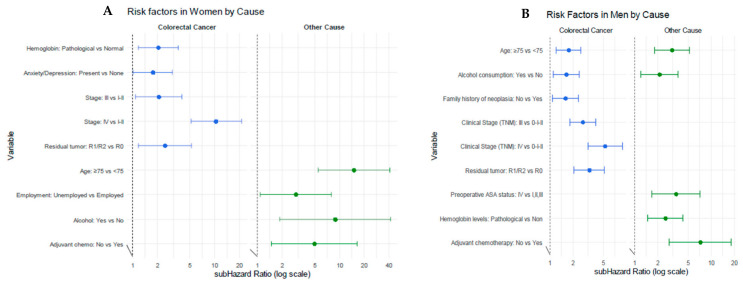
Competing Risk Model for 10-Year Mortality in Colorectal Cancer Patients by Biological Sex. (**A**) Female patients. (**B**) Male patients. The forest plot displays subdistribution hazard ratios (sHRs) from competing risk models, stratified by sex, highlighting differential associations between clinical, biological, and social factors and long-term mortality outcomes. Blue lines represent CRC-specific mortality and green lines represent non-CRC mortality.

After 10 years, more women survived, yet men reported better quality of life, highlighting the complex interplay of biological, psychosocial, and lifestyle factors, emphasizing the importance of incorporating sex-specific perspectives into survivorship care and integrating psychosocial and social determinants into long-term risk assessment (Figure 3). These subgroup findings should be interpreted as exploratory, since no formal interaction analyses were performed.

This study has several strengths, including its prospective design, long-term follow-up, and comprehensive assessment of clinical, sociodemographic, and patient-reported variables within a real-world cohort. In addition, the use of competing risk models provides a more accurate estimation of cause-specific mortality and improves the interpretability of long-term outcomes. However, several limitations should be acknowledged. Residual confounding may persist due to unmeasured factors such as nutritional status, physical activity, or social support. In addition, the categorization of several continuous variables may have reduced prognostic information and limited the assessment of potential non-linear associations. Treatment-related variables may also reflect clinical selection bias, particularly regarding the use of adjuvant chemotherapy in frail patients. Subgroup analyses by sex and tumor location should also be interpreted cautiously, as no formal interaction tests were performed and some subgroup models may have been limited by the relatively low number of events relative to the number of predictors included, potentially leading to imprecise estimates and wide confidence intervals. Furthermore, the study was initiated before the routine implementation of molecular profiling in CRC. Key biomarkers, including mismatch repair status, RAS/RAF mutations, HER2 amplification, tumor mutational burden, and other tumor-agnostic alterations, were not available for analysis [36]. Further studies integrating these molecular features are needed to refine long-term prognostic models.

## 5. Conclusions

In this 10-year population-based cohort, distinct patterns of CRC-specific and non-CRC mortality were identified, reflecting the interplay between tumor-related factors and patient vulnerability. Advanced tumor stage and residual disease were the main drivers of CRC-specific mortality, whereas non-CRC mortality was primarily associated with age, comorbidity, and markers of frailty.

Stratified analyses revealed relevant differences by tumor location and sex. Rectal cancer outcomes were more strongly influenced by systemic health and treatment-related factors, while sex-specific patterns highlighted a broader clinical risk profile in men and a greater contribution of psychosocial and social determinants in women.

These findings support the need for multidimensional risk assessment at diagnosis, integrating clinical, physiological, and psychosocial factors to improve long-term risk stratification and guide personalized survivorship strategies.

## Figures and Tables

**Table 1:** Descriptive analysis of study variables in relation to CRC-specific and other-cause mortality.

Variables	N	Alive, N = 504 ^1^	Total Deceased, N = 334	From CRC, N = 220 ^1^	From Other Causes, N = 114 ^1^	*p*-Value ^2^
Sociodemographic data						
Age	838					<0.001
<75		416 (71%)	172 (29%)	139 (81%)	33 (19%)	
≥75		88 (35%)	162 (65%)	81 (50%)	81 (50%)	
Employment status	727					0.001
Unemployed		295 (56%)	231 (44%)	150 (65%)	81 (35%)	
Employed		146 (73%)	55 (27%)	48 (87%)	7 (13%)	
(Missing)		63	48	22	26	
Clinical data						
Personal and family background	756					<0.001
No		263 (55%)	216 (45%)	135 (63%)	81 (38%)	
Yes		192 (69%)	85 (31%)	71 (84%)	14 (16%)	
(Missing)		49	33	14	19	
Smoking habit	828					0.023
Non-smoker		242 (61%)	155 (39%)	94 (61%)	61 (39%)	
Smoker		73 (69%)	33 (31%)	28 (85%)	5 (15%)	
Former smoker		185 (57%)	140 (43%)	94 (67%)	46 (33%)	
(Missing)		4	6	4	2	
Charlson Comorbidity Index	838					0.002
0		315 (68%)	150 (32%)	115 (77%)	35 (23%)	
1		122 (58%)	89 (42%)	50 (56%)	39 (44%)	
2		48 (48%)	52 (52%)	32 (62%)	20 (38%)	
3		19 (31%)	43 (69%)	23 (53%)	20 (47%)	
Preoperative analytical data						
Neutrophil-to-Lymphocyte Ratio (NLR)	642					0.031
Non-pathological (<5)		355 (62%)	213 (38%)	132 (62%)	81 (38%)	
Pathological (≥5)		34 (46%)	40 (54%)	32 (80%)	8 (20%)	
(Missing)		115	81	56	25	
Anatomical pathological data						
Tumor location	838					0.003
Right colon		130 (57%)	98 (43%)	61 (62%)	37 (38%)	
Left colon		210 (61%)	136 (39%)	80 (59%)	56 (41%)	
Rectum		164 (62%)	100 (38%)	79 (79%)	21 (21%)	
Pathological Clinical Stage (pTNM)	838					<0.001
I–II		346 (68%)	160 (32%)	78 (49%)	82 (51%)	
III		141 (55%)	117 (45%)	87 (74%)	30 (26%)	
IV		17 (23%)	57 (77%)	55 (96%)	2 (4%)	
Data on the surgical intervention						
Residual tumor after surgery	829					<0.001
R0		483 (64%)	277 (36%)	168 (61%)	109 (39%)	
R1 o R2		20 (29%)	49 (71%)	45 (92%)	4 (8.2%)	
(Missing)		1	8	7	1	
Infectious complications	838					0.1
No		450 (62%)	273 (38%)	174 (64%)	99 (36%)	
Yes		54 (47%)	61 (53%)	46 (75%)	15 (25%)	
Data related to the oncologic treatment						
Neoadjuvant chemotherapy	824					<0.001
No		427 (61%)	274 (39%)	167 (61%)	107 (39%)	
Yes		77 (63%)	46 (37%)	41 (89%)	5 (11%)	
(Missing)		0	14	12	2	
Adjuvant chemotherapy	824					<0.001
No		258 (57%)	194 (43%)	92 (47%)	102 (53%)	
Yes		246 (66%)	126 (34%)	116 (92%)	10 (7.9%)	
(Missing)		0	14	12	2	
Pre- and post-surgery radiotherapy	838					0.009
Non pre- and non post-		391 (60%)	266 (40%)	165 (62%)	101 (38%)	
Non pre- and yes post-		16 (50%)	16 (50%)	11 (69%)	5 (31%)	
Yes pre- and non post-		94 (66%)	48 (34%)	41 (85%)	7 (15%)	
Yes pre- and yes-post-		3 (43%)	4 (57%)	3 (75%)	1 (25%)	
Patient-Reported Outcomes Measures (PROMs)						
Depression (HADS-D)	719					0.023
<8		377 (63%)	221 (37%)	158 (71%)	63 (29%)	
8–10		37 (47%)	42 (53%)	21 (50%)	21 (50%)	
>10		33 (52%)	30 (48%)	19 (63%)	11 (37%)	
(Missing)		57	41	22	19	
Quality of Life (EORTC QLC-C30)	746					0.001
Low		150 (52%)	139 (48%)	87 (63%)	52 (37%)	
Medium		145 (64%)	82 (36%)	58 (71%)	24 (29%)	
High		157 (68%)	73 (32%)	52 (71%)	21 (29%)	
(Missing)		52 (57%)	40 (43%)	23 (58%)	17 (42%)	

^1^ Median (IQR) or Frequency (%). ^2^ Kruskal-Wallis rank sum test for continuous variables; Fisher’s exact test for categorical variables. Only statistically significant variables (*p* < 0.2) are shown; the rest are provided in the Appendix A Table A1. Data expressed as frequencies (row percentages).

## Data Availability

The datasets generated and/or analyzed during the current study are available from the corresponding authors upon reasonable request.

## Abbreviations

The following abbreviations are used in this manuscript:

ASA	American Society of Anesthesiologists
BMI	Body mass index
CCR	Colorectal cancer
CEA	Carcinoembryonic antigen
CI	Confidence interval
HADS	Hospital anxiety depression scale
IQR	Interquartile range
MLR	Monocytes/Lymphocytes ratio
NLR	Neutrophils/Lymphocytes ratio
PLR	Platelets/Lymphocytes ratio
PROM	Patient-reported outcome measures
SD	Standard deviation
sHR	Sub-distribution hazard ratio
SII	Systemic inflammatory index
TNM	Tumor node metastasis

## Appendix A

**Table A1:** Descriptive analysis of sociodemographic, clinical and treatment variables not significantly associated with CRC-specific and other-cause mortality.

Variables	N	Alive, N = 504 ^1^	Total Deceased, N = 334	From CCR, N = 220 ^1^	From Other Causes, N = 114 ^1^	*p*-Value ^2^
Sociodemographic data						
Sex	838					0.14
Male		307 (57%)	227 (43%)	156 (69%)	71 (31%)	
Female		197 (65%)	107 (35%)	64 (60%)	43 (40%)	
Educational level	712					0.51
Incomplete and complete primary education		315 (59%)	222 (41%)	150 (67%)	72 (33%)	
Secondary and university education		123 (70%)	52 (30%)	38 (73%)	14 (27%)	
(Missing)		66	60	32	28	
Marital status	725					0.14
Single		34 (61%)	22 (39%)	17 (77%)	5 (23%)	
Married or in a relationship		340 (64%)	193 (36%)	139 (72%)	54 (28%)	
Separated/divorced		22 (67%)	11 (33%)	7 (64)	4 (36)	
Widowed		45 (44%)	58 (56%)	33 (57%)	25 (43%)	
(Missing)		63	50	24	26	
Clinical data						
Body Mass Index	732					0.14
Underweight		1 (25%)	3 (75%)	2 (67%)	1 (33%)	
Normal weight		109 (57%)	82 (43%)	52 (63%)	30 (37%)	
Overweight		193 (62%)	120 (38%)	89 (74%)	31 (26%)	
Obesity		133 (59%)	91 (41%)	55 (60%)	36 (40%)	
(Missing)		68	38	22	16	
Alcohol consumption	784					0.77
No		418 (62%)	254 (38%)	164 (65%)	90 (35%)	
Yes		48 (43%)	64 (57%)	43 (67%)	21 (33%)	
(Missing)		38	16	13	3	
Diabetes	838					0.83
No diabetes		400 (61%)	256 (39%)	166 (65%)	90 (35%)	
Diabetes without complications		94 (57%)	72 (43%)	50 (69%)	22 (31%)	
Diabetes with complications		10 (63%)	6 (38%)	4 (67%)	2 (33%)	
Symptomatology	838					0.083
Asymptomatic		108 (21%)	23 (18%)	14 (61%)	9 (39%)	
Mild		190 (38%)	118 (38%)	87 (74%)	31 (26%)	
Moderate or severe		206 (41%)	193 (48%)	119 (62%)	74 (38%)	
Preoperative analytical data						
Platelet-to-Lymphocyte Ratio (PLR)	808					0.55
Non-pathological (<210)		481 (60%)	325 (40%)	213 (66%)	112 (34%)	
Pathological (≥210)		0 (0%)	2 (100%)	2 (100%)	0 (0%)	
(Missing)		23	7	5	2	
Systemic Immune-Inflamation Index (SII)	639					0.32
Non-pathological (<390)		137 (65%)	75 (35%)	45 (60%)	30 (40%)	
Pathological (≥390)		249 (58%)	178 (42%)	119 (67%)	59 (33%)	
(Missing)		118	81	56	25	
Preoperative data tumor markers						
CEA	836					0.88
Normal (0–5)		110 (65%)	59 (35%)	38 (64%)	21 (36%)	
Abnormal (>5)		393 (59%)	274 (41%)	181 (66%)	93 (34%)	
(Missing)		1	1	1	0	
CA 19-9	829					0.41
Normal (<37 U/mL)		297 (62%)	184 (38%)	117 (64%)	67 (36%)	
Abnormal (>37 U/mL)		203 (58%)	145 (42%)	99 (68%)	46 (32%)	
(Missing)		4	5	4	1	
Data on the surgical intervention						
ASA status	825					0.39
I, II, III		489 (62%)	302 (38%)	202 (67%)	100 (33%)	
IV		8 (24%)	26 (76%)	15 (58%)	11 (42%)	
(Missing)		7	6	3	3	
Type of intervention	838					>0.99
Scheduled/Deferred		501 (60%)	330 (40%)	217 (66%)	113 (33%)	
Urgent		3 (43%)	4 (57%)	3 (75%)	1 (25%)	
Surgical approach (main)	584					0.25
Open		13 (52%)	12 (48%)	8 (67%)	4 (33%)	
Laparoscopy		74 (49%)	76 (51%)	52 (68%)	24 (32%)	
Combined		153 (61%)	99 (39%)	53 (54%)	46 (46%)	
Others		103 (66%)	54 (34%)	33 (61%)	21 (39%)	
(Missing)		161	93	74	19	
Surgical complications	827					0.57
No		449 (61%)	290 (39%)	191 (66%)	99 (34%)	
Yes		55 (63%)	33 (38%)	20 (61%)	13 (39%)	
(Missing)		0	11	9	2	
Patient-Reported Outcome Measures (PROMs)						
Anxiety (HADS-A)	742					0.24
<8		276 (61%)	176 (39%)	125 (71%)	51 (29%)	
8–10		86 (58%)	63 (42%)	41 (65%)	22 (35%)	
>10		87 (62%)	54 (38%)	32 (59%)	22 (41%)	
(Missing)		55	41	22	19	

^1^ Median (IQR) or Frequency (%). ^2^ Kruskal-Wallis rank sum test; Fisher’s exact test.

**Table A2:** Multivariate competing risk models for 10-year CRC-specific and other-cause mortality.

		Colorectal Cancer-Specific Mortality			Other-Cause-Mortality		
		Beta (s.e.)	Hazards Ratio (95% Confidence Interval)	*p*-Value	Beta (s.e.)	HR (95% Confidence Interval)	*p*-Value
Age	<75	Reference			Reference		
	≥75	0.65 (0.15)	1.92 (1.43, 2.58)	<0.001	1.27 (0.24)	3.57 (2.19, 5.08)	<0.001
Employment status	Unemployed	-	-	-	0.82 (0.40)	2.26 (1.03, 4.98)	0.04
	Employed	-	-	-	Reference		
ASA status	I, II, III	Reference			Reference		
	IV	0.98 (0.28)	2.68 (1.54, 4.67)	<0.001	0.98 (0.34)	2.68 (1.37, 5.26)	0.004
Alcohol consumption	No	Reference			Reference		
	Yes	0.72 (0.17)	2.06 (1.45, 2.92)	<0.001	0.92 (0.27)	2.51 (1.48, 4.27)	<0.001
Hemoglobin	Non-pathologic	Reference			Reference		
	Pathologic	0.35 (0.15)	1.42 (1.07, 1.88)	0.02	0.84 (0.24)	2.33 (1.45, 3.73)	<0.001
Adjuvant chemotherapy	No	-			1.72 (0.38)	5.59 (2.65, 11.81)	<0.001
	Si	-	-	-	Reference		
Clinical stage TNM	0, I, II	Reference			-		
	III	0.92 (0.16)	2.52 (1.82, 3.49)	<0.001	-	-	-
	IV	1.97 (0.20)	7.18 (4.77, 10.82)	<0.001	-	-	-
Residual tumor after surgery	R0	Reference			-		
	R1/R2	0.99 (0.18)	2.7 (1.81, 3.9)	<0.001	-	-	-
C-index		0.76 (0.71, 0.78)				0.85 (0.80, 0.86)	

**Table A3:** Baseline characteristics of patients with colon vs. rectal cancer and corresponding *p*-values for group comparisons.

Variable	N	Total, N = 838 ^1^	Colon, N = 264 ^1^	Rectum, N = 574 ^1^	*p*-Value ^2^
Sociodemographic data					
Age	838				0.006
<75		588 (71%)	202 (77%)	386 (67%)	
≥75		250 (29%)	62 (23%)	188 (33%)	
Educational level	712				>0.99
Incomplete and complete primary education		537 (75%)	169 (75%)	368 (75%)	
Secondary and university education		175 (25%)	55 (25%)	120 (25%)	
(Missing)		97	31	66	
Marital status	725				0.35
Single		56 (7%)	23 (10%)	33 (6.7%)	
Married or in a relationship		533 (75%)	169 (73%)	364 (74%)	
Separated/divorced		33 (4%)	11 (4.8%)	22 (4.5%)	
Widowed		103 (14%)	28 (12%)	75 (15%)	
(Missing)		113	33	80	
Employment status	727				0.90
Unemployed		526 (72%)	165 (72%)	361 (72%)	
Employed		201 (28%)	64 (28%)	137 (28%)	
(Missing)		111	35	76	
Clinical data					
Body Mass Index	732				0.44
Underweight		4 (1%)	2 (1%)	2 (0.4%)	
Normal weight		191 (26%)	62 (28%)	129 (25.4%)	
Overewight		313 (43%)	99 (44%)	214 (42.1%)	
Obesity		224 (30%)	61 (27%)	163 (32.1%)	
(Missing)		106	40	66	
Personal and family background	756				0.006
No		479 (63%)	143 (57%)	336 (67%)	
Yes		277 (37%)	110 (43%)	167 (33%)	
(Missing)		82	11	71	
Vital Status after Follow-up	838				0.002
Alive		505 (60%)	164 (62%)	341 (59%)	
CCR		219 (26%)	79 (30%)	140 (24%)	
Other causes		114 (14%)	21 (8%)	93 (16%)	
Smoking habit	828				0.82
Non-smoker		397 (48%)	122 (47%)	275 (48%)	
Smoker		106 (13%)	36 (14%)	70 (12%)	
Former smoker		325 (39%)	102 (39%)	223 (39%)	
(Missing)		10	4	6	
Alcohol consumption	784				0.53
No		672 (86%)	214 (85%)	458 (86%)	
Yes		112 (14%)	39 (15%)	73 (14%)	
(Missing)		54	11	43	
Charlson Comorbidity Index	838				0.37
0		465 (55%)	151 (57%)	314 (55%)	
1		211 (25%)	62 (23%)	149 (26%)	
2		100 (12%)	36 (14%)	64 (11%)	
3		62 (8%)	15 (6%)	47 (8%)	
Diabetes	838				0.76
No diabetes		656 (78%)	210 (80%)	446 (78%)	
Diabetes without complications		166 (20%)	50 (19%)	116 (20%)	
Diabetes with complications		16 (2%)	4 (1%)	12 (2%)	
Symptomatology	838				<0.001
Asymptomatic		131 (16%)	27 (10%)	104 (18%)	
Mild		308 (37%)	139 (53%)	169 (29%)	
Moderate or severe		399 (48%)	98 (37%)	301 (52%)	
Preoperative data analytical					
Neutrophil to Lymphocyte Ratio (NLR)	642				0.006
Non-pathological (<5)		568 (88%)	165 (83%)	403 (91%)	
Pathological (≥5)		74 (12%)	33 (17%)	41 (9.2%)	
(Missing)		196	66	130	
Platelet to Lymphocyte Ratio (PLR)	808				>0.99
Non-pathological (<210)		806 (99.8%)	253 (100%)	553 (100%)	
Pathological (≥210)		2 (0.2%)	0 (0%)	2 (0.4%)	
(Missing)		30	11	19	
Systemic Immune-Inflammation Index (SII)	639				0.59
Non-pathological (<390)		212 (33%)	68 (35%)	144 (33%)	
Pathological (≥390)		427 (67%)	128 (65%)	299 (67%)	
(Missing)		199	68	131	
Hemoglobin	825				>0.99
Non-pathological (<13)		359 (43%)	94 (36%)	265 (47%)	
Pathological (≥13)		466 (57%)	166 (64%)	300 (53%)	
(Missing)		13	4	9	
Preoperative data tumor markers					
CEA	836				<0.001
Normal (0–5)		169 (20%)	18 (7%)	151 (26%)	
Abnormal (>5)		667 (80%)	245 (93%)	422 (74%)	
(Missing)		2	1	1	
Ca 19-9	829				<0.001
Normal (<37 U/mL)		481 (58%)	123 (47%)	358 (63%)	
Abnormal (>37 U/mL)		348 (42%)	138 (53%)	210 (37%)	
(Missing)		9	3	6	
Anatomical pathological data					
Pathological Clinical Stage (pTNM)	838				0.16
I–II		506 (60%)	172 (65%)	334 (58%)	
III		258 (31%)	72 (27%)	186 (32%)	
IV		74 (9%)	20 (8%)	54 (10%)	
Data on the surgical intervention					
ASA status	825				0.17
I, II, III		791 (96%)	251 (97%)	540 (95%)	
IV		34 (4%)	7 (3%)	27 (5%)	
(Missing)		13	6	7	
Type of intervention	838				0.10
Scheduled/Deferred		831 (99.2%)	264 (100%)	567 (99%)	
Urgent		7 (0.8%)	0 (0)	7 (1%)	
Surgical approach (main)	584				0.024
Open		25 (4%)	1 (10%)	24 (4%)	
Laparoscopy		150 (26%)	6 (60%)	144 (25%)	
Combined		252 (43%)	1 (10%)	251 (44%)	
Others		157 (27%)	2 (20%)	155 (27%)	
(Missing)		254	254	0	
Residual tumor after surgery	829				<0.001
R0		760 (92%)	223 (85%)	537 (95%)	
R1 o R2		69 (8%)	39 (15%)	30 (5.3%)	
(Missing)		9	2	7	
Surgical complications	827				<0.001
No		739 (89%)	209 (81%)	530 (93%)	
Yes		88 (11%)	49 (19%)	39 (7%)	
(Missing)		11	6	5	
Infectious complications	838				0.42
No		723 (86%)	224 (85%)	499 (87%)	
Yes		115 (14%)	40 (15%)	75 (13%)	
Data related to the oncologic treatment					
Neoadjuvant chemotherapy	824				<0.001
No		701 (85%)	145 (56%)	556 (98%)	
Yes		123 (15%)	112 (44%)	11 (2%)	
(Missing)		14	7	7	
Adjuvant chemotherapy	824				<0.001
No		452 (55%)	119 (46%)	333 (59%)	
Yes		372 (45%)	138 (54%)	234 (41%)	
(Missing)		14	7	7	
Pre- and post-surgery radiotherapy	838				<0.001
Non pre- and non post-		657 (78%)	98 (37%)	559 (97%)	
Non pre- and yes post-		32 (4%)	21 (8%)	11 (2%)	
Yes pre- and non post-		142 (17%)	138 (52%)	4 (1%)	
Yes pre- and yes-post-		7 (1%)	7 (3%)	0 (0%)	
Data reported outcomes measures (PROMS)					
Anxiety (HADS-A)	742				0.19
<8		452 (61%)	136 (58%)	316 (62%)	
8–10		149 (20%)	56 (24%)	93 (18%)	
>10		141 (19%)	41 (18%)	100 (20%)	
(Missing)		96	31	65	
Depression (HADS-D)	719				0.97
<8		598 (81%)	189 (81%)	409 (81%)	
8–10		79 (11%)	24 (10%)	55 (11%)	
>10		63 (8.5%)	20 (9%)	43 (8%)	
(Missing)		98	31	67	
EORTC QLC-C30	746				0.042
Low		289 (39%)	77 (33%)	212 (41%)	
Medium		227 (30%)	84 (36%)	143 (28%)	
High		230 (31%)	73 (31%)	157 (31%)	
(Missing)		92	30	62	

^1^ Median (IQR) or Frequency (%). ^2^ Wilcoxon rank sum test; Pearson’s Chi-squared test; Fisher’s exact test.

**Table A4:** Risk Factors for Mortality in Patients with Colon Tumors.

		Colorectal Cancer-Specific Mortality			Other-Cause-Mortality		
		Beta (s.e.)	Hazards Ratio (95% Confidence Interval)	*p*-Value	Beta (s.e.)	HR (95% Confidence Interval)	*p*-Value
Age	<75	Reference			Reference		
	≥75	0.58 (0.25)	1.80 (1.09, 2.94)	0.02	2.11 (0.51)	8.29 (3.07, 22.34)	<0.001
Alcohol consumption	No				Reference		
	Yes				1.31 (0.53)	3.70 (1.31, 10.46)	0.014
Sex	Male	0.63 (0.27)	1.88 (1.1, 3.21)	0.02			
	Female	Reference					
Residual tumor after surgery	R0	Reference					
	R1/R2	0.72 (0.28)	2.05 (1.17, 3.57)	0.01			
Postoperative infectious complications	No	Reference					
	Yes	0.92 (0.28)	2.52 (1.46, 4.34)	<0.001			
Clinical Stage (TNM)	0, I, II	Reference					
	III	0.69 (0.26)	1.99 (1.19, 3.33)	<0.001			
	IV	1.59 (0.35)	4.92 (2.46, 9.83)	<0.001			
Adjuvant chemotherapy	No				1.95 (0.76)	7.03 (1.58, 31.14)	0.01
	Yes				Reference		
C-index	0.73 (0.65, 0.78)				0.87 (0.78, 0.90)		

**Table A5:** Risk Factors for Mortality in Patients with Rectal Tumors.

		Colorectal Cancer-Specific Mortality			Other-Cause-Mortality		
		Beta (s.e.)	Hazards Ratio (95% Confidence Interval)	*p*-Value	Beta (s.e.)	Hazards Ratio (95% Confidence Interval)	*p*-Value
Age	<75	Reference			Reference		
	≥75	0.69 (0.22)	2.01 (1.3, 3.11)	0.001	1.41 (0.24)	4.09 (2.53, 6.63)	<0.001
Charlson Index	0				Reference		
	1				0.65 (0.27)	1.93 (1.34, 3.28)	0.14
	2				1.11 (0.31)	3.05 (1.64, 5.66)	<0.001
	3				0.93 (0.34)	2.24 (1.31, 4.92)	0.005
Preoperative ASA status	I, II, III	Reference			Reference		
	IV	1.04 (0.52)	2.84 (1.01, 7.98)	0.04	1.08 (2.97)	2.97 (1.51, 5.84)	0.001
Alcohol consumption	No	Reference			Reference		
	Yes	0.88 (0.26)	2.42 (1.45, 4.06)	<0.001	0.96 (0.28)	2.61 (1.5, 4.56)	<0.001
Residual tumor after surgery	R0	Reference					
	R1 or R2	1.62 (0.29)	5.05 (2.81, 9.09)	<0.001			
Clinical Stage (TNM)	0, I, II	Reference					
	III	0.97 (0.26)	2.65 (1.58, 4.46)	<0.001			
	IV	2.65 (0.32)	14.22 (7.52, 26.88)	<0.001			
Adjuvant chemotherapy	No				1.44 (0.40)	4.24 (1.9, 9.48)	<0.001
	Yes				Reference		
Hemoglobin	Non-pathologic	Reference			Reference		
	Pathologic	0.57 (0.22)	1.77 (1.15, 2.74)	0.01	0.60 (0.23)	1.83 (1.16, 2.88)	0.009
Lymphocyte-to-Neutrophil Ratio (NLR)	No-pathologic	Reference					
	Pathologic	0.83 (0.28)	2.21 (1.26, 3.85)	0.005			
Adjuvant Chemotherapy	No	1.13 (0.55)	3.11 (1.045, 9.27)	0.04			
	Yes	Reference					
C-index		0.81 (0.75, 0.83)			0.85 (0.80, 0.87)		

**Table A6:** Risk Factors for Mortality in Women.

		Colorectal Cancer-Specific Mortality			Other-Cause-Mortality		
		Beta (s.e.)	Hazards Ratio (95% Confidence Interval)	*p*-Value	Beta (s.e.)	Hazards Ratio (95% Confidence Interval)	*p*-Value
Age	<75	-			Reference		
	≥75	-	-	-	2.71 (0.51)	14.98 (5.48, 40.91)	<0.001
Current employment	Unemployed	-			1.07 (0.50)	2.93 (1.08, 7.95)	0.03
	Employed	-	-	-	Reference		
Hemoglobin levels	Non-pathological	Reference					
	Pathological	0.72 (0.28)	2.06 (1.18, 3.61)	0.01			
Anxiety (HADS-A) and Depression (HADS-D)	No axiety or depression	Reference			-		
	Anxiety and/or Depression	0.56 (0.28)	1.76 (1.01, 3.07)	0.04	-	-	-
Alcohol consumption	No	-			Reference		
	Yes	-	-	-	2.18 (0.79)	8.85 (1.86, 42.06)	0.006
Clinical Stage (TNM)	0, I, II	Reference			-		
	III	0.73 (0.33)	2.08 (1.08, 4.00)	0.02	-	-	-
	IV	2.34 (0.35)	10.39 (5.14, 21.25)	<0.001	-	-	-
Residual tumor after surgery	R0	Reference			-		
	R1 o R2	0.91 (0.37)	2.49 (1.18, 5.23)	0.01	-	-	-
Adjuvant chemotherapy	No	-			1.59 (0.61)	4.93 (1.48, 16.43)	0.009
	Yes	-	-	-	Reference		
C-index		0.74 (0.66, 0.79)			0.88 (0.79, 0.92)		

**Table A7:** Risk Factors for Mortality in Men.

		Colorectal Cancer-Specific Mortality			Other-Cause-Mortality		
		Beta (s.e.)	Hazards Ratio (95% Confidence Interval)	*p*-Value	Beta (s.e.)	Hazards Ratio (95% Confidence Interval)	*p*-Value
Age	<75	Reference			Reference		
	≥75	0.56 (0.18)	1.75 (1.21, 2.53)	0.002	1.12 (0.26)	3.08 (1.82, 5.19)	<0.001
Preoperative ASA status	I, II, III	-			Reference		
	IV	-	-	-	1.24 (0.37)	3.46 (1.67, 7.16)	<0.001
Hemoglobin levels	Non-pathological	-			Reference		
	Pathological	-	-	-	0.92 (0.27)	2.52 (1.48, 4.3)	<0.001
Alcohol consumption	No	Reference			Reference		
	Yes	0.49 (0.20)	1.64 (1.10, 2.42)	0.01	0.75 (0.28)	2.12 (1.21, 3.71)	0.008
Family history of neoplasia	No	0.46 (0.19)	1.59 (1.08, 2.35)	0.01	-		
	Yes	Reference			-	-	-
Adjuvant chemotherapy	No	-			1.97 (0.47)	7.19 (2.83, 18.31)	<0.001
	Yes	-	-	-	Reference		
Clinical Stage (TNM)	0, I, II	Reference			-		
	III	0.99 (0.19)	2.69 (1.82, 3.98)	<0.001	-	-	-
	IV	1.66 (0.26)	5.29 (3.16, 8.86)	<0.001	-	-	-
Residual tumor after surgery	R0	Reference			-		
	R1 o R2	1.18 (0.23)	3.26 (2.05, 5.17)	<0.001	-	-	-
C-index		0.74 (0.68, 0.77)			0.84 (0.79, 0.87)		
